# Assessing the Validity and Reliability of a Markerless Motion Capture System for Sagittal-Plane Gait Range of Motion

**DOI:** 10.7759/cureus.99875

**Published:** 2025-12-22

**Authors:** Tsuyoshi Ohmura, Satoshi Kojima, Toshiki Azuma, Kei Narui, Sayaka Futatsuya, Saki Tsuchida, Tomoyuki Maruo, Katsushi Suwa

**Affiliations:** 1 Department of Rehabilitation, Keiju Kanazawa Hospital, Kanazawa, JPN; 2 Rehabilitation Science, Graduate School of Rehabilitation, Kinjo University, Hakusan, JPN; 3 Rehabilitation Medicine, Kinjo University, Hakusan, JPN; 4 Department of Rehabilitation, Kanazawa Izumigaoka Watanabe Clinic, Kanazawa, JPN; 5 Department of Rehabilitation, Tanakamachi Onsen Care Center, Kanazawa, JPN; 6 Department of Rehabilitation, Kanazawa Minami Care Center, Kanazawa, JPN; 7 Rehabilitation, Nara Child Therapeutic School BELIEVE, Yamato-Koriyama, JPN

**Keywords:** artificial intelligence, biomechanics, gait analysis, inertial measurement unit, lower limb kinematics, markerless motion capture, range of motion, reliability, smartphone application, validity

## Abstract

Introduction: Optical three-dimensional (3D) motion capture systems and inertial measurement unit (IMU)-based systems provide accurate kinematic measurements, yet both remain costly and require complex setups. Artificial intelligence (AI)-driven markerless systems that operate with a single smartphone offer a practical alternative. In this study, we evaluated the validity and reliability of a markerless motion capture system (SPLYZA Motion; SPLYZA Inc., Shizuoka,* *Japan) for sagittal-plane gait analysis relative to an IMU-based 3D system (Ultium Motion; Noraxon Inc., Scottsdale, AZ, USA).

Methods and analysis: Twenty healthy adults walked at a self-selected pace, and hip, knee, and ankle joint angles were recorded simultaneously using both systems. Joint angles were time-normalized, and waveform characteristics and peak values were compared. Associations were assessed using Spearman’s rank correlation (ρ), agreement was examined using intraclass correlation [ICC(3,1)] with 95% confidence intervals (CIs), and proportional and fixed bias were evaluated using linear regression and Wilcoxon signed-rank tests. Agreement was further assessed using Bland-Altman analysis.

Results: Significant positive correlations were observed for the hip (ρ = 0.913, p < 0.01), knee (ρ = 0.833, p < 0.01), and ankle (ρ = 0.721, p < 0.01). ICCs demonstrated excellent agreement for the hip (0.914; 95% CI: 0.906-0.920) and knee (0.934; 95% CI: 0.929-0.940) and good agreement for the ankle (0.723; 95% CI: 0.701-0.743). Root-mean-square error (RMSE) values (mean ± SD) were 7.73 ± 3.32° (hip), 7.48 ± 3.40° (knee), and 6.38 ± 2.44° (ankle). Bland-Altman plots indicated small biases for the hip and knee, with wider limits for the ankle near end-range positions.

Conclusion: SPLYZA Motion showed accuracy and reliability comparable to Ultium Motion within clinically acceptable limits. Minor ankle-related biases were small enough to avoid affecting clinical interpretation. Given its low cost and portability, this system provides a practical option for gait assessment in routine clinical and research environments.

Clinical relevance: Minimal setup AI-based markerless motion capture enables a feasible clinical gait analysis.

## Introduction

Quantitative gait analysis, which supplies objective information on movement characteristics, has traditionally relied on optical three-dimensional (3D) motion capture systems using reflective markers and infrared cameras, or on inertial sensor-based systems equipped with inertial measurement units (IMUs). Optical systems are regarded as the gold standard in motion-analysis research because they can accurately track marker trajectories and reconstruct joint kinematics in 3D space [1,2]. In contrast, IMU-based systems record linear acceleration and angular velocity through accelerometers and gyroscopes, integrating these inputs to estimate segment orientations and joint ranges of motion. Although numerous studies have validated their accuracy and reproducibility [3-6], both optical and IMU-based systems remain expensive, time-consuming, and dependent on complex setups and calibration procedures that limit their routine use in many clinical environments [7].

In recent years, AI-driven markerless motion-analysis applications using monocular smartphone or tablet cameras have emerged as practical alternatives. These tools are lightweight, portable, and cost-effective, enabling motion analysis without markers or sensor attachments. Previous studies have reported high correlations between markerless and conventional systems for relatively simple spatiotemporal gait parameters such as walking speed and step length [8]. However, moderate correlations have been documented for some joint angles and phases of the gait cycle, likely reflecting occlusion effects and limitations in two-dimensional (2D)-to-3D estimation, which continue to pose accuracy challenges. A recent systematic review summarized these observations and noted that although markerless systems can yield reliable results for many metrics, their precision in estimating joint angles remains inconsistent across systems and movements [9].

Despite these developments, direct comparisons between markerless motion capture and IMU-based systems remain limited. Most existing studies have examined optical versus markerless comparisons or hybrid sensor fusion approaches [10,11]. Consequently, validating markerless motion capture against an IMU-based reference standard is both clinically relevant and scientifically meaningful because it reflects the practical feasibility of gait analysis in rehabilitation settings. SPLYZA Motion (SPLYZA Inc., Shizuoka, Japan), a commercially available iOS-based markerless motion-analysis application, performs 3D motion tracking using a single camera. Several recent case reports and research studies have incorporated this application [12,13], demonstrating its expanding use in clinical and sports rehabilitation. Nonetheless, its quantitative validity for evaluating lower-limb joint angles and range of motion during walking remains insufficiently established. Sagittal-plane limb range of motion constitutes a fundamental component of gait assessment, particularly in patients with hip pathology and total hip replacement [14]. In knee osteoarthritis, sagittal-plane knee kinematics have been associated with important clinical outcomes such as altered loading patterns and structural progression [15,16]. Rehabilitation studies have also monitored changes in sagittal-plane knee kinematics to assess intervention effects, supporting their role as clinically meaningful outcome measures [17,18]. Establishing the validity and reliability of SPLYZA Motion may therefore clarify its utility as a cost-effective and practical option for quantitative gait assessment, thereby facilitating objective evaluation of motor performance and treatment outcomes and supporting clinical decision-making in rehabilitation practice. Accordingly, the objective of this study was to evaluate the validity and reliability of SPLYZA Motion for estimating sagittal-plane hip, knee, and ankle joint angles during level walking in healthy adults, using Ultium Motion (Noraxon Inc., Scottsdale, AZ, USA), an IMU-based system, as the reference standard.

Portions of this work were presented at the 52nd Annual Meeting of the Japanese Society for Clinical Biomechanics, held in Kyoto, Japan, in November 2025.

## Materials and methods

Ethics

The experimental design of this study was approved by the Research Ethics Committee of Kinjo University (Approval No. 2024-07). Written informed consent was obtained from all participants before enrollment.

Study design and subjects

Twenty healthy adult men participated in this study (mean age: 20.5 ± 0.76 years; height: 172.0 ± 4.4 cm; weight: 61.9 ± 6.4 kg; BMI: 20.9 ± 2.2 kg/m2). An a priori power analysis was conducted using G*Power version 3.1 (Heinrich Heine University, Düsseldorf, Germany). Based on the primary outcome of detecting the intraclass correlation coefficient [ICC(3,1)] (expected ICC = 0.85; null ICC = 0.60; α = 0.05; power = 0.80), the minimum required sample size was 13 participants. To ensure sufficient power and accommodate potential exclusions, 20 participants were recruited. All volunteers were free from musculoskeletal or neurological disorders that could influence gait.

Experimental setup

**Figure 1:**
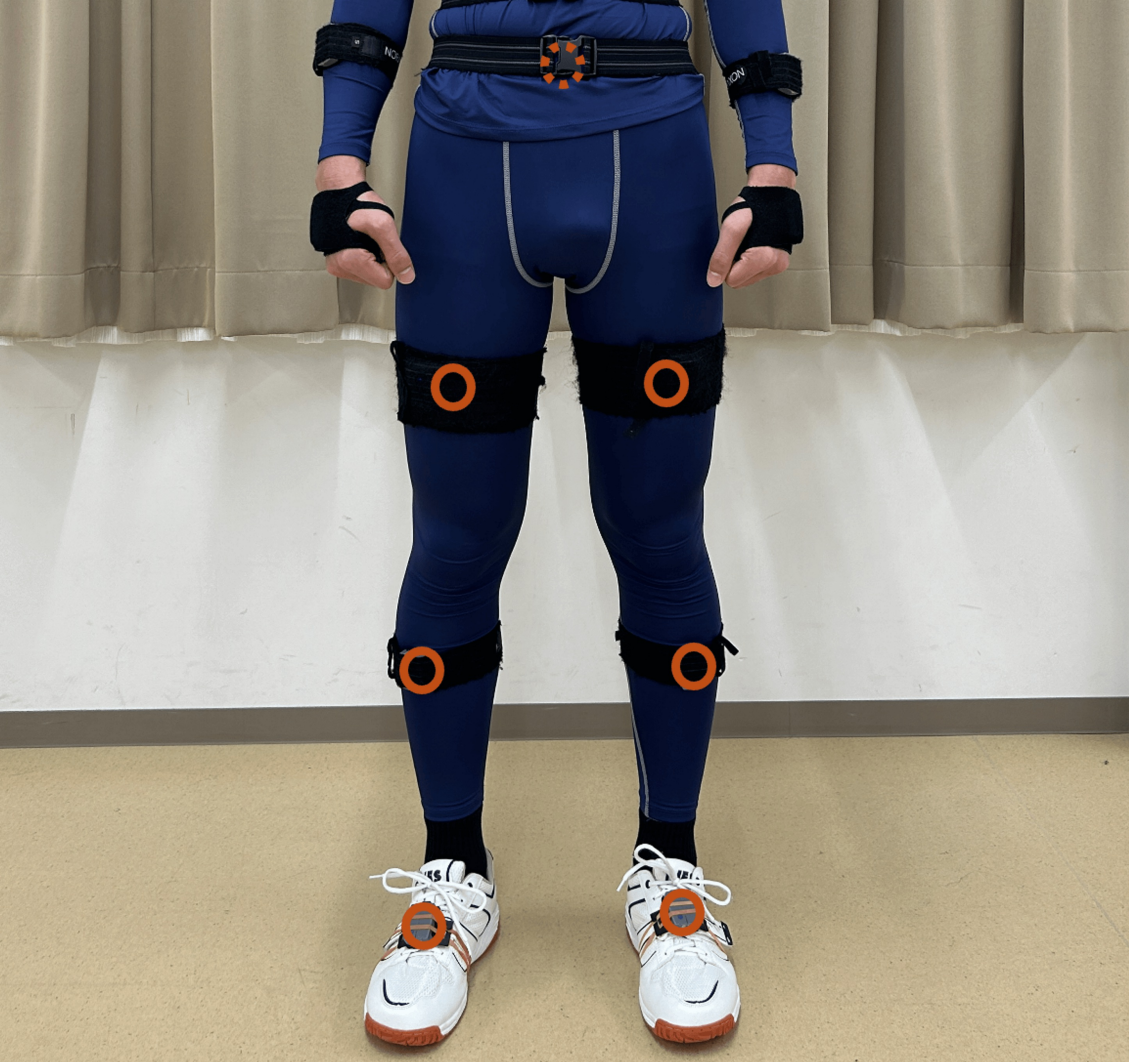
Placement of inertial sensors. Sensors were attached to the sacrum, thigh, lower leg, and foot using customized straps.

Participants wore form-fitting leggings, identical socks, and shoes selected according to foot measurements (JES-005; Japan Educational Shoe Council, Japan). To maintain consistency, one examiner completed all shoe fittings. Seven Ultium Motion inertial sensors were attached at standardized anatomical locations: the midpoint of the superior sacral border, the anterior thigh at two-thirds of the femoral length proximally, the anterior shank at one-third of the tibial length distally, and the point 10 cm anterior to the midpoint between the medial and lateral malleoli on both feet. Sensors were secured using specialized straps (Figure 1). Before data collection, the Ultium Motion system was calibrated according to the manufacturer’s standard static calibration protocol with the participant standing in an upright anatomical position. Each participant walked along a 10-meter walkway at a comfortable self-selected pace.

**Figure 2:**
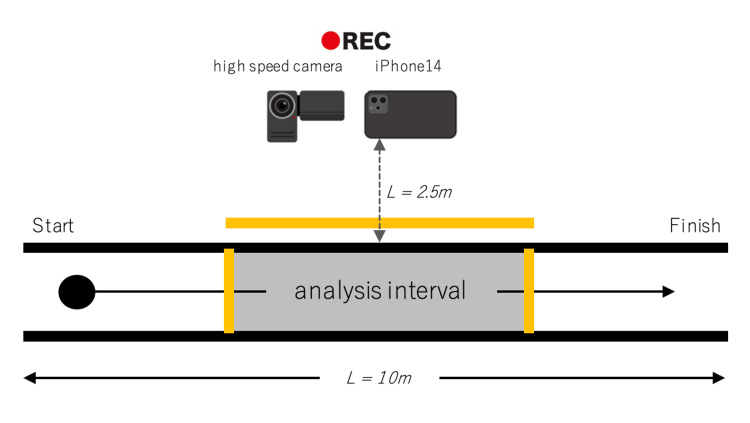
Gait analysis setup (plan view, schematic) The analysis interval was marked with colored tape. The iPhone 14 was positioned 2.5 m lateral to the midpoint of this interval, and the high-speed camera was placed directly beside it.

**Figure 3:**
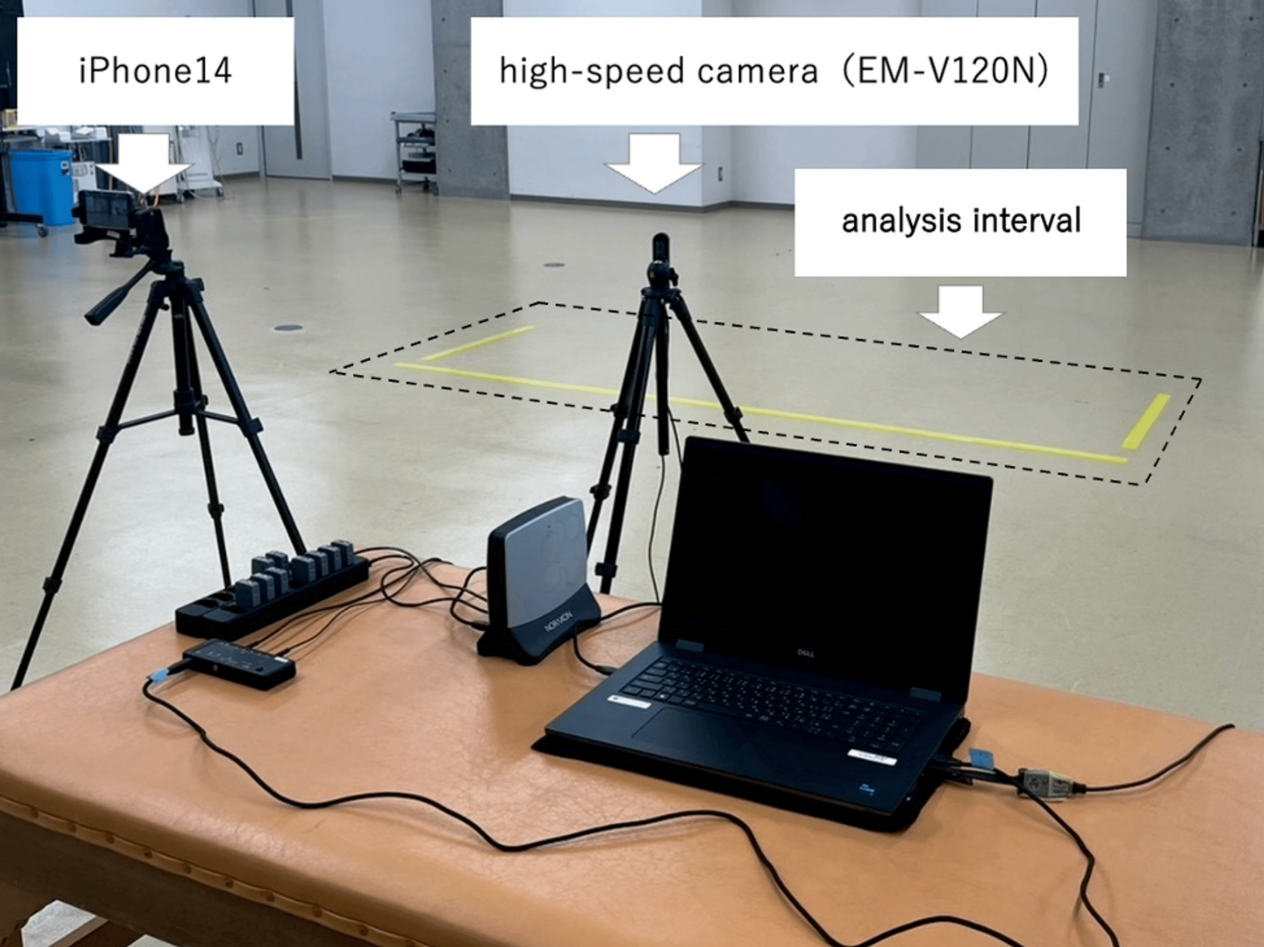
Gait analysis setup (lateral view, photograph) Shown are the iPhone 14 used for SPLYZA Motion analysis and the high-speed camera used for Ultium Motion recording. Both cameras were mounted on tripods and photos were obtained at a standardized height of 1.0 m.

During the walking trials, a sagittal-plane video was recorded with a high-speed camera (EM-V120N; Noraxon Inc.) at 120 frames per second (fps) to validate event identification in IMU-referenced data extraction. At the same time, a second sagittal-plane video for SPLYZA Motion analysis was recorded using an iPhone 14 (Apple Inc., Cupertino, CA, USA) at 720p resolution and 240 fps. All signals and videos were collected simultaneously during the same walking trial (Figures 2, 3). The smartphone was mounted on a tripod, positioned centrally, 2.5 m from the walkway and at a height of 1.0 m. The iPhone’s built-in horizontal grid and level indicators were activated to maintain appropriate camera alignment. Colored tape was placed at both ends of the analysis segment and kept within the field of view to confirm and maintain parallel alignment between the walkway and imaging plane. All trials were performed under uniform indoor lighting (approximately 500 lux) to ensure that no visual obstruction entered the camera’s field of view. To minimize variability, each participant completed one familiarization trial followed by two valid trials in a fixed sequence. In all cases data from the second trial were used for statistical analysis.

Data processing

**Figure 4:**
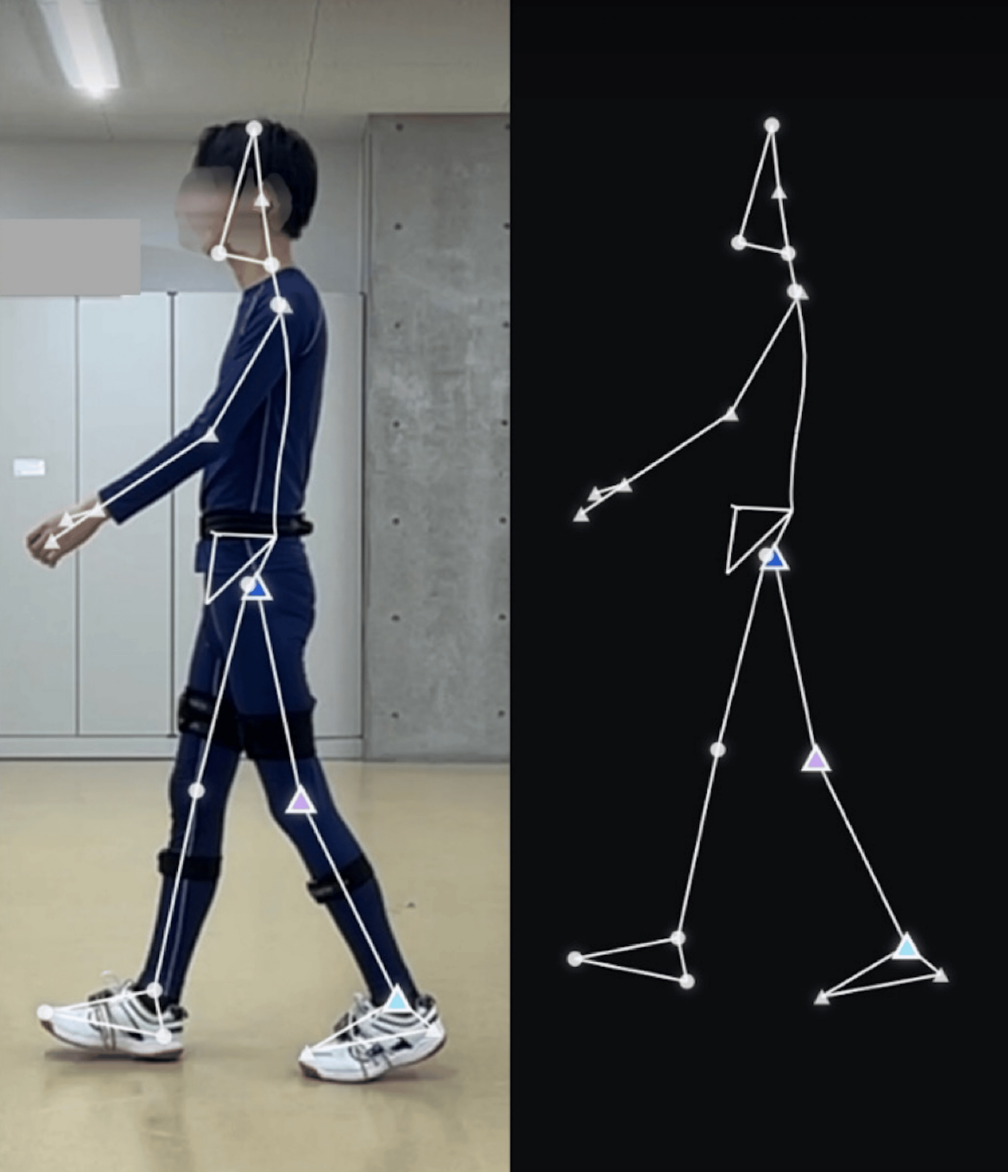
Screenshot of the SPLYZA Motion analysis interface. The image displays a sagittal-plane analysis as obtained using SPLYZA Motion. Shown are points featuring the hip, knee, and ankle.

During this study, we analyzed two consecutive ipsilateral gait cycles within the mid-walkway steady-state segment. Ultium Motion data were processed using myoRESEARCH 3 software (Noraxon Inc.), and smartphone videos were analyzed with the SPLYZA Motion application (Figure 4). Both systems produced sagittal-plane joint angles for the hip, knee, and ankle. Ultium Motion data were sampled at 200 Hz, and SPLYZA Motion videos were recorded at 240 fps, with joint angles computed frame by frame.

Heel strike (HS) and toe-off (TO) events were annotated manually, frame by frame, on sagittal videos. These videos were obtained using slow-motion footage (240 fps) from an iPhone 14 (for the SPLYZA-referenced extraction) and high-speed camera recordings (EM-V120N; 120 fps) for the IMU-referenced extraction. We then defined HS as the first visible contact of the heel with the floor, and defined TO as the final visible detachment of the toe from the floor.

For each subject, we defined two consecutive left-limb gait cycles by three successive left heel strikes (i.e., LHS1, LHS2, and LHS3). Next, Ultium Motion and SPLYZA Motion datasets were segmented according to identical gait-event intervals (i.e., LHS1-LHS2 and LHS2-LHS3). Finally, data characterizing these two cycles were concatenated and time-normalized to a 0-100% gait cycle. Joint-angle waveforms were then exported in comma-separated values (CSV) format for subsequent statistical analysis.

Statistical analysis

All statistical analyses were conducted using R version 4.2.1 (R Foundation for Statistical Computing, Vienna, Austria). Inter-device reproducibility was assessed using the ICC(3,1) with 95% confidence intervals (95% CI). Values were interpreted as poor (<0.40), fair (0.40-0.59), good (0.60-0.74), or excellent (≥0.75). Normality of data was assessed using a Shapiro-Wilk test.

The strength of association between systems was summarized with Spearman’s rank correlation coefficient; these values were categorized as moderate (0.40-0.69), strong (0.70-0.89), or very strong (≥0.90). Proportional error was assessed using simple linear regression, with SPLYZA Motion values as the dependent variable and Ultium Motion values as the independent variable. We reported the slope (β1) and coefficient of determination (R2).

Fixed error was evaluated by dividing the two gait cycles (i.e., Cycles 1 and 2) into stance and swing phases and comparing peak maximum and minimum joint angles within each phase using a Wilcoxon signed-rank test. Waveform similarity was then quantified using root-mean-square error (RMSE) computed over 101 time-normalized data points (0-100% gait cycle). These values were summarized as the group mean ± SD for each joint. For visualization, device-wise mean ± SD joint-angle trajectories were overlaid across the normalized cycle.

Bland-Altman analysis was also used to evaluate agreement between systems. For each joint and each metric (i.e., peak maximum and peak minimum angles), results were summarized for Cycle 1 and Cycle 2. The average of the two methods and their difference (SPLYZA minus Ultium) were used to compute the bias (mean difference) and 95% limits of agreement, which were plotted. Proportional bias was examined by regressing the difference on the average; when detected, regression-adjusted limits of agreement were reported. The significance level was set at α = 0.05. Finally, all visualizations (i.e., Bland-Altman plots and waveform overlays) were generated using Python version 3.14.0 (Python Software Foundation, Wilmington, DE, USA).

## Results

Next, the reproducibility between Ultium Motion and SPLYZA Motion was evaluated using ICC(3,1). Recorded ICC values were as follows: 0.914 (95% CI: 0.906-0.920) for the hip joint, 0.934 (95% CI: 0.929-0.940) for the knee joint, and 0.723 (95% CI: 0.701-0.743) for the ankle joint (Table 1). These results indicated excellent agreement for the hip and knee joints and good agreement for the ankle joint.

**Table 1:** Intraclass correlation coefficients [ICC(3,1)] and 95% confidence intervals (CI). Shown are intraclass correlation coefficients [ICC(3,1)] with 95% confidence intervals for each joint. Qualitative interpretation: excellent (≥0.75) and good (0.60–0.74).

Joint	ICC(3,1)	95% CI	Reliability
Hip	0.914	0.906–0.920	Excellent
Knee	0.934	0.929–0.940	Excellent
Ankle	0.723	0.701–0.743	Good

Spearman’s rank correlation coefficients further demonstrated significant positive associations between the two systems for all joints: hip (ρ = 0.913, p < 0.01), knee (ρ = 0.833, p < 0.01), and ankle (ρ = 0.721, p < 0.01) (Table 2). The hip joint showed a very strong correlation, whereas the knee and ankle demonstrated strong correlations. RMSE values (mean ± SD) were 7.73 ± 3.32° (hip), 7.48 ± 3.40° (knee), and 6.38 ± 2.44° (ankle) (Table 3), reflecting waveform consistency within approximately 10° across joints.

**Table 2:** Spearman’s rank correlation coefficients (ρ) for the Ultium Motion and SPLYZA Motion methods. Spearman’s rank correlation coefficients (ρ) between Ultium Motion and SPLYZA Motion measurements. Data are shown for each joint. p-values correspond to tests of ρ ≠ 0. Qualitative interpretation: very strong (≥0.90) and strong (0.70–0.89).

Joint	Spearman’s ρ	p-value	Interpretation
Hip	0.913	<0.01	Very strong
Knee	0.833	<0.01	Strong
Ankle	0.721	<0.01	Strong

**Table 3:** RMSE values between Ultium Motion and SPLYZA Motion recorded for each joint. Waveform error, expressed as root-mean-square error (RMSE; mean ± SD, °), between Ultium Motion and SPLYZA Motion over the time-normalized gait cycle (0–100%; 101 data points).

Joint	RMSE [°]	± SD
Hip	7.73	± 3.32
Knee	7.48	± 3.40
Ankle	6.38	± 2.44

To assess proportional bias, simple linear regression analyses were performed with Ultium Motion as the independent variable and SPLYZA Motion as the dependent variable. The regression slopes were 0.082 (R² = 0.038, p < 0.001) for the hip, 0.066 (R² = 0.032, p < 0.001) for the knee, and 0.084 (R² = 0.011, p < 0.001) for the ankle (Table 4). Although the slopes were statistically significant, the very low coefficients of determination indicated that mean joint angle explained only a negligible portion of the variance in inter-device differences. Thus, any detected proportional bias is unlikely to be clinically meaningful.

**Table 4:** Linear regression analysis results assessing the proportional bias between the SPLYZA Motion and Ultium Motion values. Proportional bias was evaluated using simple linear regression, with SPLYZA Motion values as the dependent variable and Ultium Motion values as the independent variable. Shown are the regression slope (β₁), its p-value, and the coefficient of determination (R²) for each joint examined.

Joint	Slope	R²	p-value
Hip	0.082	0.038	<0.001
Knee	0.066	0.032	<0.001
Ankle	0.084	0.011	<0.001

Fixed error was evaluated using the Wilcoxon signed-rank test to compare maximum and minimum joint angles during the stance and swing phases of two consecutive gait cycles. Several maximum and minimum angles of the hip joint showed significant differences across both cycles. For the knee joint, only the maximum stance-phase angle in the first gait cycle reached significance. In contrast, the ankle joint showed multiple significant differences for both maximum and minimum angles during stance and swing phases across both cycles (Table 5), indicating that inter-device discrepancies were most prominent near the extremes of dorsiflexion and plantarflexion.

**Table 5:** Wilcoxon signed-rank test results for maximum and minimum joint angles. Wilcoxon signed-rank tests were conducted to compare peak maximum (Max) and minimum (Min) joint angles. Data shown for the stance and swing phases across two consecutive gait cycles. Reported measurements indicate median values. U = Ultium Motion; S = SPLYZA Motion. *p < 0.05, **p < 0.01, ***p < 0.001.

Joint	Peak (Max/Min)	Cycle	Gait phase	U, median (°)	S, median (°)	p-value
Hip	Max	1	Stance phase	23.229	26.971	>0.05
Hip	Min	1	Stance phase	-21.118	-14.083	<0.001***
Hip	Max	1	Swing phase	23.229	26.971	<0.05*
Hip	Min	1	Swing phase	-13.580	-8.580	<0.01**
Hip	Max	2	Stance phase	21.280	25.291	<0.05*
Hip	Min	2	Stance phase	-18.807	-12.387	<0.001***
Hip	Max	2	Swing phase	24.254	31.528	<0.001***
Hip	Min	2	Swing phase	-9.370	1.341	<0.001***
Knee	Max	1	Stance phase	45.696	52.424	<0.001***
Knee	Min	1	Stance phase	0.542	0.725	>0.05
Knee	Max	1	Swing phase	60.512	58.515	>0.05
Knee	Min	1	Swing phase	2.020	2.066	>0.05
Knee	Max	2	Stance phase	50.646	56.733	>0.05
Knee	Min	2	Stance phase	1.793	1.968	>0.05
Knee	Max	2	Swing phase	67.819	66.590	>0.05
Knee	Min	2	Swing phase	2.504	3.884	>0.05
Ankle	Max	1	Stance phase	13.522	17.392	<0.05*
Ankle	Min	1	Stance phase	-8.028	-4.856	<0.01**
Ankle	Max	1	Swing phase	8.309	16.614	<0.001***
Ankle	Min	1	Swing phase	-11.255	-3.550	<0.001***
Ankle	Max	2	Stance phase	12.753	16.658	<0.05*
Ankle	Min	2	Stance phase	-10.713	-3.851	<0.01**
Ankle	Max	2	Swing phase	6.816	10.597	<0.05*
Ankle	Min	2	Swing phase	-10.123	-6.027	<0.001***

**Figure 5:**
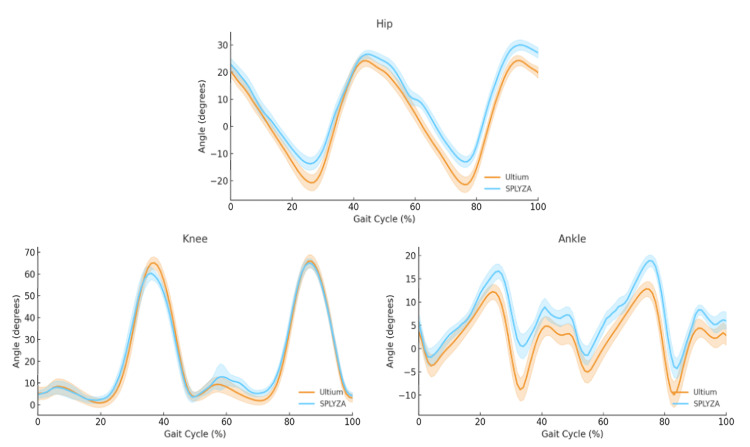
Device-wise mean ± SD joint-angle waveforms obtained over the normalized gait cycle (0–100%). Data obtained for the hip, knee, and ankle. Shown are group-averaged joint-angle waveforms for the Ultium Motion (orange) and SPLYZA Motion (blue) systems, with shaded areas indicating ± SD. Data were time-normalized to a 0–100% heel-strike-to-heel-strike gait cycle (n = 20). Positive values reflect flexion and dorsiflexion.

Waveform overlay comparisons revealed strong visual similarity between Ultium Motion and SPLYZA Motion for the hip and knee joints. The ankle joint displayed broadly comparable patterns; however, SPLYZA Motion tended to yield slightly higher recorded values than Ultium Motion throughout the gait cycle, with larger deviations appearing at the end ranges of plantarflexion and dorsiflexion (Figure 5).

**Figure 6:**
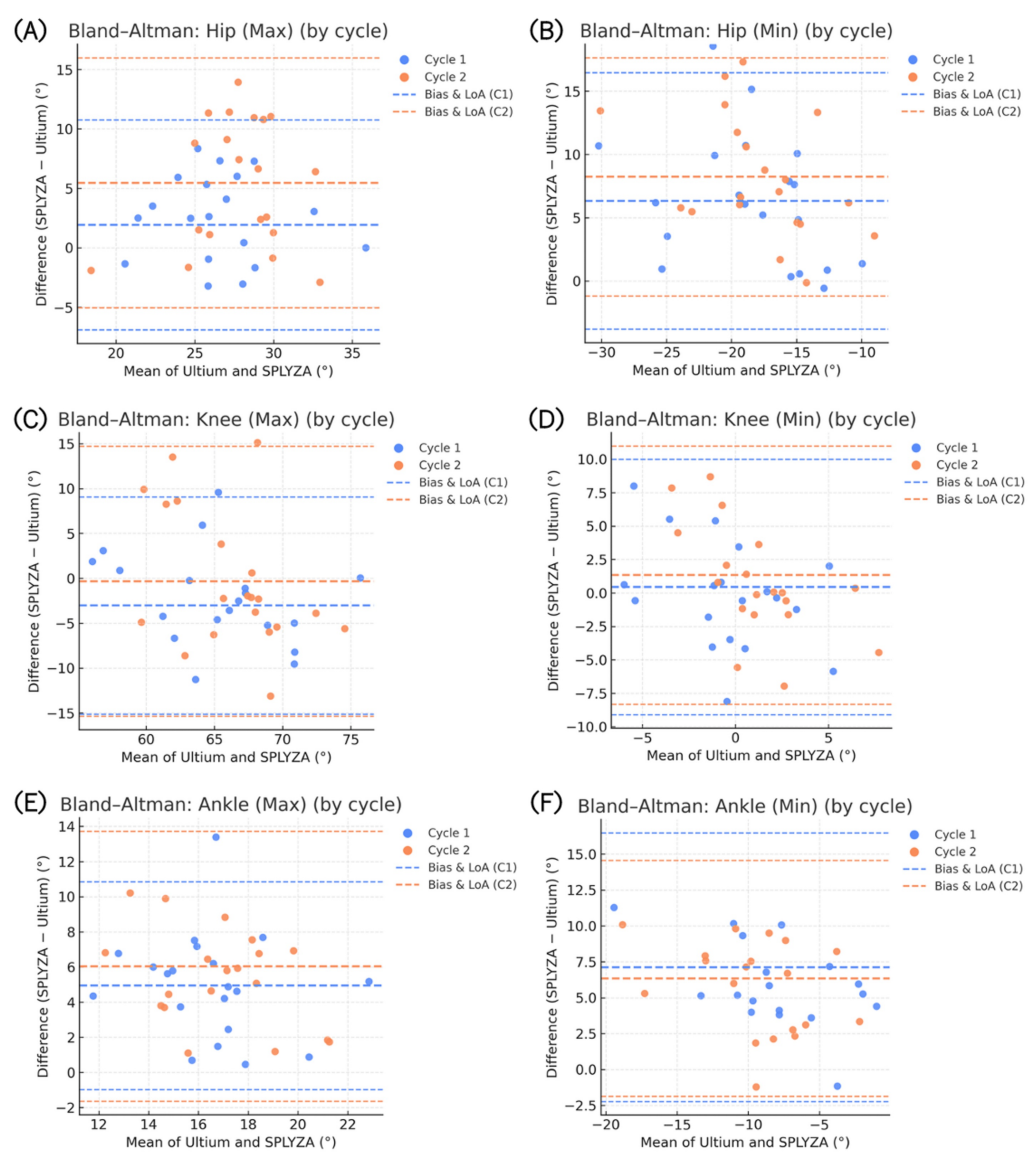
Bland–Altman plots of gait kinematics (panels A–F). Shown are the following panels: (A) hip maximum, (B) hip minimum, (C) knee maximum, (D) knee minimum, (E) ankle maximum, and (F) ankle minimum angles. The X-axis presents the mean of SPLYZA Motion and Ultium Motion values, and the Y-axis shows their difference (SPLYZA − Ultium). Each point represents data from Cycle 1 or Cycle 2, and the dashed lines denote the bias (mean difference) and the 95% limits of agreement (LoA) for each cycle. Positive values indicate SPLYZA > Ultium. Abbreviations: max, maximum; min, minimum; LoA, limits of agreement.

Next, we conducted Bland-Altman analyses (Figure 6A-6F). When examined separately, Cycle 1 and Cycle 2 showed a minor positive bias (SPLYZA > Ultium) for maximum values and a minor negative bias (SPLYZA < Ultium) for minimum values in the hip joint. The limits of agreement (LoA) were well within a clinically acceptable range. For the knee joint, maximum and minimum biases were near zero, with little difference between cycles. In contrast, the ankle joint exhibited a clear positive bias, particularly for minimum values reflecting end-range plantarflexion, and demonstrated wider LoA than the other joints.

As shown in Figure 6, the bias and LoA for Cycle 1 and Cycle 2 overlapped substantially, and no meaningful discrepancies were observed between trials. These results indicate that systematic error was minimal for the hip and knee joints, whereas ankle-related error tended to be comparatively greater at the end ranges of motion.

## Discussion

In this study, we sought to evaluate the validity and reliability of lower-limb joint-angle measurements in the sagittal plane during walking by comparing SPLYZA Motion with Ultium Motion using data from 20 healthy adults. Overall, the two systems showed high agreement in hip, knee, and ankle kinematics during gait. A proportional error analysis revealed that SPLYZA Motion showed a directional bias at the ankle and tended to report greater dorsiflexion and smaller plantarflexion values near end-range positions. In contrast, the coefficients of determination were minimal across all joints, indicating that the regression models had limited explanatory value. Although the Bland-Altman regressions produced statistically significant slopes, the extremely low R² values indicated that the mean joint angle contributed only minimally to the variability in measurement differences. Given the large number of observations (n = 2,020 data points per device), the statistical significance likely reflects high analytical power rather than a clinically meaningful trend. Thus, proportional bias in this dataset should be interpreted as negligible in practice. Collectively, inter-device differences appear to stem mainly from fixed (constant) errors and random variation rather than angle-dependent proportional bias.

With regard to fixed errors, significant discrepancies were identified across several hip and ankle metrics, whereas differences in the knee were more limited. At the ankle, SPLYZA Motion tended to report greater dorsiflexion and smaller plantarflexion than Ultium Motion, indicating a directional bias in end-range estimation. Bland-Altman analysis supported these observations: biases were small and LoA for the hip and knee remained within practically acceptable ranges. The ankle, however, showed a positive bias and wider LoA, especially at end-range positions. Previous reports have shown that even optical motion capture systems can yield lower-limb joint-angle discrepancies of several degrees, sometimes approaching 5°, due to operator factors such as marker placement and soft-tissue artifact [1]. Likewise, comparisons of IMU-based methods with optoelectronic reference systems have reported sagittal-plane hip, knee, and ankle flexion/extension errors generally below 8°, describing these values as comparable to clinically used optoelectronic systems [19]. The RMSE and maximum/minimum differences observed here were within these ranges. Furthermore, studies comparing markerless and optical systems have found differences of similar magnitude in lower-limb sagittal-plane kinematics [20-23], supporting the present results. Consequently, SPLYZA Motion demonstrated accuracy broadly comparable to established systems for estimating sagittal-plane hip, knee, and ankle joint angles during level walking in healthy adults.

From an implementation standpoint, optical systems remain costly and require complex installation, whereas IMU-based systems require multiple sensors and calibration. In contrast, SPLYZA Motion uses a single monocular camera and AI-based processing, requiring minimal equipment and preparation. The errors observed in this study were within previously reported inter-system variations, and high consistency was demonstrated for the hip and knee joints. These findings suggest that SPLYZA Motion is suitable for screening, longitudinal monitoring, and feedback applications in clinical practice.

However, fixed and proportional errors may persist for certain ankle parameters, particularly near end-range positions and other extrema. Longitudinal comparisons within the same device should therefore be prioritized, and device-specific optimization of cutoff thresholds, combined with comprehensive evaluation using metrics such as RMSE and segment mean values, is recommended. Even so, joint- and motion-specific limitations remain, reflecting the challenges inherent in deriving 3D estimations from 2D imagery. Appropriate methodological considerations, including systematic error correction when comparing devices, are therefore warranted.

In particular, ankle-related errors may influence assessments of gait disorders and the fitting of ankle orthoses, so interpretation should account for device-specific systematic biases. Future integration of real-time feedback and automated error-correction algorithms may further enhance the clinical utility of AI-based markerless motion capture systems.

Limitations

This study has several limitations. First, participants were restricted to healthy young males, which limits generalizability to females, older adults, and clinical populations. Second, the analysis focused solely on the sagittal plane using a single monocular camera and therefore did not capture multiplanar kinematics or more demanding motor tasks (e.g., stair ambulation or directional changes). Third, differences in video frame rates and device sampling frequencies may have introduced minor synchronization discrepancies. Fourth, contralateral lower-limb joints were not examined, preventing evaluation of side-to-side asymmetries. Finally, only two consecutive gait cycles per participant were analyzed, which may have limited the extent to which natural variability in gait kinematics was captured. Future studies should recruit more diverse and clinically relevant cohorts, incorporate multiplanar assessments that include the contralateral limb, and determine the responsiveness of this method to clinically meaningful changes, such as those arising from therapeutic interventions.

## Conclusions

SPLYZA Motion demonstrated strong correlations and high agreement with Ultium Motion for hip, knee, and ankle joint angles during gait, with accuracy remaining within clinically acceptable limits. Although small fixed and proportional errors were detected near end-range positions, particularly at the ankle, their magnitude was clinically negligible and did not limit practical use.

Given its low cost, portability, and minimal setup demands, SPLYZA Motion offers a practical and user-friendly option for supporting gait analysis in routine clinical settings. For clinical interpretation, within-device longitudinal comparisons should be emphasized, and waveform-level measures (e.g., RMSE) should be considered alongside peak joint angles. Overall, this study provides new evidence supporting the clinical utility of AI-based, markerless motion-analysis systems for quantitative gait assessment.
